# A multiphase recruitment approach to enroll Latinas diagnosed with cervical cancer into a qualitative study at an academic medical center in the Pacific Northwest

**DOI:** 10.1017/cts.2025.85

**Published:** 2025-05-19

**Authors:** Cirila Estela Vasquez Guzman, Yareli Cornejo Torres, Meredith Zauflik

**Affiliations:** 1 Department of Family Medicine, Oregon Health & Science University, Portland, OR, USA; 2 Richmond Family Health Center, Department of Family Medicine, Oregon Health & Science University, Portland, OR, USA; 3 Oregon Clinical & Translational Research Institute, Oregon Health & Science University, Portland, OR, USA

**Keywords:** Participant recruitment, Latinos, diversity, enrollment, oncology

## Abstract

Recruitment of diverse populations into research studies continues to be a challenge. There remains a gap in knowledge and practice on how to best engage with and recruit diverse populations, specifically among Latinos who account for 11% of research participants nationally. Our study focused on Latinas with pre-cervical and cervical cancer in the Pacific Northwest. Our research team took a multilevel approach to diversify recruitment and enrollment processes, focused on methods within healthcare, community-based, and paid media advertisements. This article shares strategies and lessons learned that helped increase participant diversity, meet enrollment goals, and expand relationships with community-based organizations.

## Introduction

Recruitment of diverse populations into research studies continues to be a challenge. Study teams often grapple with enrolling a representative study population and knowing how to best do that [1–3]. Specifically among Latinos, according to a recent study, they account for about 18.5% of the U.S. population, however, only represent about 11% of research participants [4]. This is in stark contrast to the overrepresentation of Non-Hispanic Whites, who account for 62% of the population, but 76.3% of participants [4].

There is a growing interest among researchers in how to better engage with and recruit underrepresented populations [5, 6]. While many researchers working with Latino communities are adopting creative recruitment strategies, many of these strategies rely solely on collaborating with a Promotora (Community Health Worker) or community organization to diversify recruitment [6, 7]. These community partners are effective at building trust, however, reliance on them limits recruitment reach. Thereby, establishing trust, building capacity, and using additional methods remain a gap in recruitment strategies, impacting enrollment.

Our study “Taking a Life Course Perspective to Examine Critical Points of Intervention for Latina Women Diagnosed with Cervical Cancer,” an Oregon Health & Science University (OHSU) Institutional Review Board approved study, created an evolving multi-method, multi-phase recruitment strategy to recruit Latinas diagnosed with pre-cervical and cervical cancer, utilizing a broad and diverse approach that combined traditional and community-based approaches. Recruitment success was measured not only on enrollment numbers, but relationships established and increased community awareness of cervical cancer. Our aim of this article is to discuss our recruitment strategy, outcomes and successes, and key lessons to help others create and utilize a diverse range of practices in their engagement and recruitment strategies.

## Materials and methods

### Study overview

There is a large disparity between cancer incidence and mortality rate between racial/ethnic minority and non-minority populations, including Latinos [8]. Cervical cancer is the fourth most common cancer diagnosis in Oregon and has larger incidence rates among Latinas vs. Non-Hispanic Whites [9]. This study leveraged a qualitative approach to understand the experiences and structural-level factors that shape Latinas cervical cancer trajectories [10]. Inclusion criteria included Latinas with a cervix, aged 35 years or older, diagnosed with pre-cervical or cervical cancer within the last ten to fifteen years, living in the Pacific Northwest.

### The team

An important aspect of the study design was intentionally structuring the research team to be reflective of the study population, which enabled us to bring lived experience and understanding of known barriers to connecting with and recruiting this population. Thirteen of our fourteen team members were women. Of these women, eleven identify as women of color, eight were bilingual in Spanish, including the principal investigator, three master-level research assistants, two Latina undergraduate students, three Latina analysts, one Black regulatory specialist, and a Latina 1.5 generation (someone born in one country, yet raised in another) who coordinated recruitment and study coordination. Two team members identify as white women, one served as an analyst and the other a recruitment specialist from the Oregon Clinical and Translational Research Institute (OCTRI), the NIH CTSA program hub at OHSU. One team member identified as Latino male and served as the videographer.

### Multi-level recruitment approach

Recruitment occurred between November 2021 and July 2024. We began with a community advisory board (CAB) in May 2022, seeking recommendations and specific cultural and community strategies. Based on this, we began recruitment through community engagement focused on the Latinos population in the Portland metro area. In December 2022, our team then engaged OCTRI’s recruitment specialist, who worked with the team to create additional tailored methods based on successes and best practices for the study population and enrollment goals. The recruitment specialist provided ongoing consultation and support, meeting with the study team bi-monthly to adjust the recruitment strategy based on recruitment efforts and outcomes. Our overall recruitment strategy became an evolving, multi-method, multi-phase approach, focused within healthcare, community, and paid media advertisements, combining both traditional and community-based approaches. Once implemented, methods were ongoing and overlapped with each other.

## Results

**Figure 1.**
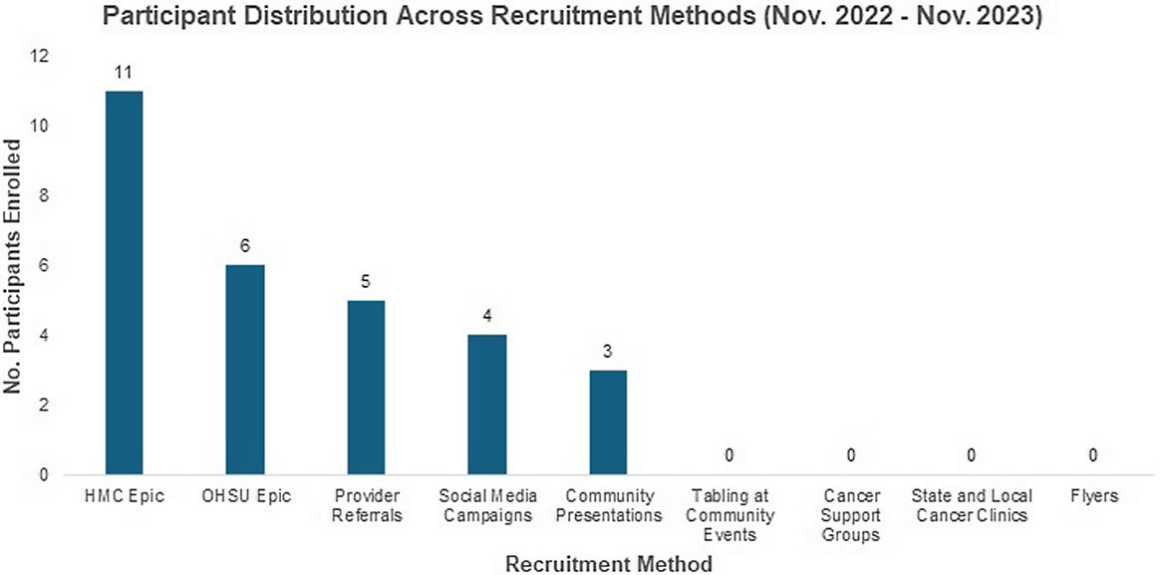
Participant distribution across recruitment methods. Legend: This graph numerically distributes participants enrolled into our study by the recruitment method by which they were enrolled.

As of July 2024, 51 participants are enrolled. This exceeded initial enrollment goals of 35–40 individuals. Participants were also reflective of the diversity within Latinos – 72.5% spoke Spanish, 66.7% reported low English proficiency, ∼50% were formally or currently undocumented, and 33% reported limited formal K-12 education. Our results include the first 29 participants enrolled before November 2023 (Figure 1). Results of participants enrolled after this date (*N* = 22) were inconsistently tracked due to limited staffing and were a result of previously implemented methods. Success of methods was defined by our team to include not only enrollees produced but also community relationships established and increased community education on cervical cancer.

### Traditional healthcare recruitment

We utilized Epic recruitment tools within the electronic health records of OHSU and OHSU’s affiliate site Hillsboro Medical Center (HMC), collaborating with an informatician to build the search query based on eligibility criteria. Once identified, our recruitment coordinator contacted individuals by phone. For OHSU, between May 2022 and May 2023, we obtained a list of 106 individuals, 26 were eligible post pre-screening, and six enrolled. Through HMC, which has a large percentage of Latinos patients, we obtained a list of 76 individuals, 63 were eligible after pre-screening, and 11 enrolled.

We also engaged providers, with the PI working with obstetrician/gynecologists (OB-GYN), who referred patients to the study. Providers were given postcards and fliers to share with those they identified. Two enrolled into the study. Additionally, four clinic shadowing visits by the PI with a clinician on our CAB, where the PI directly met with their patients, resulted in three more participants enrolling.

Despite building a search query in Epic that matched our study criteria, the results were not always accurate (e.g., age). Adding in extra time to re-review each result was necessary. Additionally, not everyone was aware of or understood their diagnosis, though previously shared with them by their provider, so more time was needed in the recruitment script to ensure they understood it.

### Community-based recruitment

We then transitioned our efforts toward community-oriented outreach, attending various community tabling events between early 2022 through May 2023, identified through formal and informal processes. We attended seven total events – six community events throughout the Portland Metro area and one on the Oregon coast. During these events, we shared over 100+ postcards and fliers. Although we did not enroll anyone as a direct result of these events, we were able to establish relationships with over 20 community health centers, community-based clinics, and Latinos nonprofit organizations, which are ongoing with the intent of lasting partnerships (Table 1).

**Table 1.** Community organization demographics

	Organization Type					Geographic Location				Organization Size				Language Offered		
Organization	Health Center	Community Center	Nonprofit	Government	Mixed Types	Urban	Suburban	Rural	Statewide	Grassroots	Midlevel	Regional	National/Local Office	English Only	Spanish Only	Bilingual
1			x					x				x				x
2			x			x							x	x		
3			x						x	x						x
4		x					x				x					x
5			x			x				x				x		
6			x			x						x				x
7			x						x				x	x		
8	x					x					x			x		
9				x					x				x			x
10			x			x						x				x
11				x		x							x			x
12				x					x				x			x
13			x			x							x	x		
14			x			x					x					x
15				x		x							x			x
16			x			x					x					x
17				x		x							x	x		
18				x		x							x	x		
19			x						x				x			x
20		x					x			x					x	
21					x	x							x			x
22	x								x				x			x
23			x				x			x						x
24					x				x				x			x
25				x		x							x			x
26					x				x				x			x
**Total**	**2**	**2**	**12**	**7**	**3**	**14**	**3**	**1**	**8**	**4**	**4**	**3**	**15**	**7**	**1**	**18**
**Percentage of Organizations**	**7.7%**	**7.7%**	**46.2%**	**26.9%**	**11.5%**	**53.8%**	**11.5%**	**3.8%**	**30.8%**	**15.4%**	**15.4%**	**11.5%**	**57.7%**	**26.9%**	**3.8%**	**69.2%**

Legend: This table details the partnerships we made with community organizations as part of this study and important descriptive characteristics for each one, including type of organization, geographic location, organization size, and language offered. Organization number is their respective study coding, which was done to respect the privacy of our community partners.

Our team also connected with cancer support groups and treatment clinics across the region. We contacted 12 support groups, sharing social media language, flyers, and newsletter articles. No participants were enrolled through these efforts. We reached out to 98 local and state treatment clinics from January 2023 to May 2023, mailing informational packages that included an introductory letter, fliers, and postcards. No participants were enrolled.

The PI also gave seven presentations in community-based settings. First, we presented in English and Spanish to community pathway navigators and community promotoras at three different events. Next, an English language presentation was given to the Knight Cancer Center & OCTRI Community Liaison Program, who have six community representatives across the state. Through a local community organization, a Spanish language presentation was given to promotoras. Additionally, we presented at an annual bilingual community-based conference put on by a local nonprofit organization. Three participants were enrolled through these efforts.

Based on anecdotal evidence received by our community partners, through our community engagement efforts, we were able to build visibility and trust in the community, along with raising cancer awareness. Additionally, through our funding mechanism, we were able to provide dedicated staff time, which enabled these time and labor intensive, but critically important, outreach efforts.

### Paid media advertisement recruitment

Our team’s two undergraduate students, both first generation aspiring OB-GYN Latinas, created a recruitment flyer with a family member in mind as the audience. All the fliers were bilingual (English and Spanish), in plain language, at or below 6^th^ grade reading level, and included a variety of formats such as single flyers and tear tabs. Two decisions were made that tailored them to our participant population. First, we featured a picture of the PI, who is a woman of color, wearing a traditional Oaxacan yellow dress. Second, we used bright colors and featured an image of a Latina provider and patient speaking to one another. Flyers were posted across 26+ locations, including Latinos businesses, schools, public spaces, OHSU research desks, and clinics. Additionally, around 200+ fliers were shared with our networks. No enrollment was connected to the flyers, though they received a lot of attention and comments from community partners and potential participants.

Study information was shared through OHSU’s Women’s Health Research Unit (WHRU) and Center for Women’s Health Clinic newsletter and social media channels, along with OHSU’s main social media channel. The newsletter was in both English and Spanish. The social media campaign was created with the OHSU social media team. We shared study information through WHRU’s Facebook (2.4k followers), Instagram (979 followers), and newsletter (5,752 subscribers). OHSU shared via Facebook (59K followers) and Instagram (18.7K followers). The paid campaign was live from January 2023 to June 2023, with two rounds of advertising. Another round occurred between January 2024 and May 2024. Through the first round, 62,112 individuals in English and 77,808 in Spanish viewed the ad. During the second round, 54,879 individuals in English and 61,393 in Spanish viewed the ad. We enrolled four participants from these methods.

Working with our undergraduate students, who are from outside research, brought new insights, creativity, and ideas to the recruitment process. Furthermore, collaborating with OHSU communication experts increased study visibility within and outside our institution.

## Discussion

Based on our study disease focus and target population, we found it necessary to diversify our recruitment strategy, mixing traditional healthcare and community-based recruitment approaches. This allowed us to not only meet enrollment goals but also goals of community partnerships and increased disease awareness. Building and maintaining relationships with community-based organizations and key trusted community members was foundational to developing trust, expanding enrollment, and benefits future engagement and research [6, 7]. When engaging with a historically disadvantaged population, it was critical to have existing relationships with organizations within the community, to not only build visibility and trust but also be positioned to connect participants with these organizations for needs outside the study, helping add value to the community and strengthening relationships.

Adopting an evolving, dynamic recruitment approach and a representative team was also vital to our recruitment success. Our recruitment approach was structured to remain flexible and responsive to feedback from our CAB and recruitment specialist, continually adjusting or implementing methods. Additionally, having a representative study team was critical to our ability to approach and engage with the community [1]. A study team of mainly women, majority identifying as women of color from marginalized communities, allowed us to better understand, engage with, and add familiarity and trust for our participants by having a team who reflected them.

A major limitation to this study was that staffing levels changed, impacting bandwidth and ability for certain methods, such as community tabling events. A related limitation is the time needed for community engagement based on the community-based organization or stakeholder’s staffing and bandwidth, which can impact available methods and/or lengthen recruitment timelines.

Our findings of the importance of having an evolving multi-method recruitment approach, combining traditional and community-based methods informs next steps. This includes working with our network of community organizations to disseminate study results, ensuring participants and community members know the impact of their efforts. Additionally, we will offer Spanish language presentations focused on women’s health, helping to increase education of cervical cancer and research opportunities among Latinas. This work will help to address disparities in research participation and ensure the advancement of research responsive to growing concerns around equity and inclusion.
